# Semen Quality of Rasa Aragonesa Rams Carrying the *FecXR* Allele of the *BMP15* Gene

**DOI:** 10.3390/ani10091628

**Published:** 2020-09-11

**Authors:** José Alfonso Abecia, Ángel Macías, Adriana Casao, Clara Burillo, Elena Martín, Rosaura Pérez-Pé, Adolfo Laviña

**Affiliations:** 1 University Institute of Research in Environmental Sciences of Aragon (IUCA), University of Zaragoza, Miguel Servet, 177, 50013 Zaragoza, Spain; adriana@unizar.es (A.C.); rosperez@unizar.es (R.P.-P.); 2 National Association of Rasa Aragonesa Breeders (ANGRA), Cabañera Real, s/n, 50800 Zuera, Spain; angel@rasaaragonesa.com (Á.M.); aabecia0@gmail.com (C.B.); jabecia1@alumno.uned.es (E.M.); adolfo@rasaaragonesa.com (A.L.)

**Keywords:** *BMP15*, ram, semen

## Abstract

**Simple Summary:**

It has been demonstrated that the ovine gene *BMP15* presents a mutation in the Rasa Aragonesa Spanish sheep breed, which has been called *FecXR*. In heterozygosis, ewes exhibit a variable increase in the ovulation rate, producing 0.35 additional lambs per birth and, in homozygosis, sterility. Since the importance of carrying this polymorphism in rams has not been studied, sperm quality and fertility of rams carrying the *FecXR* mutation of the ovine gene *BMP15* has been determined, comparing semen quality, testicle characteristics, and fertility rate of rams presenting or not the allele. FecXR rams exhibited a higher masal motility and a higher proportion of rapid sperm than did non-carrier rams; however, no differences in scrotal circumference or testicular length and diameter were found, although FecXR rams produced a higher proportion of pregnant ewes after artificial insemination. Thus, it seems that the *FecXR* allele creates high-quality semen and improves some sperm parameters in this breed, making these males especially valuable for artificial insemination to produce prolific ewes, when wild-type, non-carrier ewes, are inseminated.

**Abstract:**

The *FecXR* mutation is a variant of the ovine gene *BMP15* in the Rasa Aragonesa breed. Information on the physiological importance of carrying the *FecX* polymorphism in rams is limited. The aim of this study was to compare semen quality, testicle characteristics, and fertility rate of rams that carry the *FecXR* allele. Rams (*n* = 15) were either FecXR allele carriers (*n* = 10) or non-carriers, wild type (++) (*n* = 5). FecXR rams exhibited higher mass motility (*p* < 0.05), proportion of rapid sperm (*p* < 0.05), and a lower proportion of slow sperm (*p* < 0.0001) than did ++ rams. The presence of the *FecXR* allele was not associated with mean scrotal circumference or testicular length and diameter, although season had a significant (*p* < 0.05) effect on these traits. Genotype (*p* < 0.05) and season (*p* < 0.01) had a significant effect on mean fertility rate, FecXR rams had a higher proportion of pregnant ewes than did ++ rams (*p* < 0.05). In conclusion, the *FecXR* allele produced high-quality semen throughout the year, and corresponded with an improvement in some sperm parameters, particularly, mass motility and the proportion of rapid sperm.

## 1. Introduction

Several mutations in genes of the transforming growth factor-beta (TGF-β) superfamily have positive effects on ovulation rate and litter size; e.g., FecB or Bone Morphogenetic Protein (BMP) R1B, FecX or BMP15, and FecG or GDF9 (for a review [1]). Galloway et al. [2] identified a mutation in the *BMP15* gene, that introduced a stop codon on the X chromosome, which prevents the normal translation of the protein encoded by this gene and, subsequently, demonstrated its effect on the ovulation rate in a population of the Inverdale (*FecXI*) sheep breed. The mutation is sex-linked because it is located in the non-recombinant region of the X chromosome and, therefore, males can have one copy of the gene, only, but females can be hetero or homozygous for the mutation (review [3]). A male carrier transmits the mutation to all of his daughters but to none of his sons, and heterozygous females pass on the mutation to, on average, half of their offspring. However, homozygous females are sterile because they do not develop ovarian follicles correctly.

Since the discovery of the mutation in the Inverdale breed, mutations in *BMP15* have been identified in other prolific breeds including Belclare and Cambridge (*FecXB*), Hanna (*FecXH*), Galway (*FecXG*), Lacaune (*FecXL*), Rasa Aragonesa (*FecXR*), Grivette (*FecXGr*), and Olkuska (*FecXO*) [2,4,5,6,7,8,9]. In all, the mechanism of action is similar (amino acid substitutions, deletions, or stop codons), and all have received the same name (*FecX*) and the first initial of the breed in which it was discovered because the phenotypic effects are similar; i.e., in heterozygosis, a variable increase in ovulation rate and, in homozygosis, sterility [10].

The *FecXR* mutation is a variant of the ovine gene *BMP15* in the Rasa Aragonesa breed. Rasa Aragonesa is one of the most important meat sheep breeds in Spain, where there are about 1.1 million head, and 360,000 are registered in the Stud Book of the National Association of Rasa Aragonesa Breeders (ANGRA) [11]. Mean litter size is 1.2–1.5 lambs/birth [12], and ANGRA is developing a genetic improvement program that includes prolificacy as one of the important objectives. *FecXR* has been included in the selection scheme under the commercial denomination “Gen ANGRA Santa Eulalia”, and there are >5000 ewes that carry this mutation. The allele has been used to increase prolificacy in Rasa Aragonesa sheep through artificial insemination (AI) of wild type, non-carrier ewes, which are used to disseminate the allele across those farms interested in improving litter size. The positive effect of *FecXR* on prolificacy is well known; viz., 0.35 additional lambs per birth [11], which has increased cost effectiveness and profits.

Most of the studies on the expression of *FecXR* have involved female sheep and information on the physiological importance of the *FecXR* polymorphism in males, particularly, rams, is limited. Studies on *BMP15* in rams have investigated tissue expression pattern in rams that differ in fecundity [13], fertility rate [14], and the influence of the *FecB* genotype on semen attributes [15]. The aim of this study was to compare the semen quality, testicle characteristics, and fertility rate, through AI, of Rasa Aragonesa rams that carry the *FecXR* allele during different seasons, so that it is hypothesized that the efficiency of AI using these particular rams may be improved.

## 2. Materials and Methods

### 2.1. Animals

Rams were housed at CERSYRA (Regional Centre for Animal Selection and Reproduction) in Zaragoza, Spain (41°N), and were breeding males for AI in the stud book of ANGRA. Inseminations were performed on commercial farms by veterinarians of ANGRA. Approval from the Ethics Committee of the University of Zaragoza was not a prerequisite for this study. The study met the Spanish Policy for Animal Protection RD1201/05, which meets the European Union Directive 2010/63 on the protection of animals used for experimental and other scientific purposes.

Fifteen adult Rasa Aragonesa rams (age: 5.7 ± 2.8 yr) used in the study were either FecXR allele carriers (*n* = 10) or non-carriers, wild type (++) (*n* = 5). The laboratory procedures (DNA extraction, polymerase chain reaction (PCR) amplification prior to sequencing, DNA sequencing and analysis) after they had been exposed the localization of the allele are described by Monteagudo et al. [8].

### 2.2. Semen Collection and Analyses

Rams were housed together and were fed to meet their maintenance requirements. Throughout the year, semen samples (96 per ram) were collected twice a week [16], starting at 9:00 am, in an artificial vagina at 35–40 °C lubricated with petroleum jelly. Each collection day, a routine, simplified semen analysis was performed that included concentration measured by spectrophotometry (AccRead, IMV Technologies, L’Aigle, France) (1:400 dilution in saline solution plus 0.2% glutaraldehyde), volume (ml), measured in a graduated collection tube, and mass motility estimated by optical microscopy at 100× magnification and scored from 0 to 5. Once per month, the proportion of static sperm, total motile (TM) sperm, non-progressive (NPM) and progressive (PM), and motile sperm subpopulations (rapid, medium, or slow sperm) were measured in a computer-assisted sperm analysis (CASA) using ISAS software (Integrated Semen Analysis System, Proiser, Paterna, Valencia, Spain). Semen sample processing and motility and viability assessment followed the method of Palacín et al. [17]. Briefly, before motility or viability analysis, 200 × 10^6^ sperm/mL semen samples were mixed and re-diluted to a final concentration of 50 × 10^6^ sperm/mL using INRA 96 (IMV Technologies, L’Aigle, France) extender. An Olympus BX40 microscope under 100× magnifications, provided with heated stage set at 37 °C, was used to estimate sperm motility. The grade of the forward progression (fast progressive, slow progressive and motile but not progressive) determined on the TM sperm were recorded. Sperm with curvilinear velocity (VCL) ≥ 75 m/sg and straightness (STR) ≥ 80% were considered rapid progressive and with VCL < 5 m/sg and STR ≥ 80% slow progressive.

Thereafter, the semen was diluted (INRA 96) and put into French mini-straws for AI (0.25 mL, 300 × 106 spermatozoa/mL).

### 2.3. Testicular Measurements

Once every month, scrotal circumference (SC) (pulling the testes firmly down into the lower part of the scrotum and placing a measuring tape into a loop around the greatest diameter over the scrotum), length (placing the fixed arm of a caliper at the proximal end and the sliding arm at the distal end of the testes) and diameter (placing one arm of a caliper at the medial aspect and the other at the lateral aspect of the testes, at the point of maximum width) of each testicle, were determined. Testicular length (TL) and diameter (TD) were calculated as the mean of both testicles.

### 2.4. Artificial Inseminations (AI)

In the 12 months of the study, 1412 AI were performed on 29 farms. To synchronize estrus, vaginal sponges containing 30 mg of fluorogestone were applied for 12 d. At pessary withdrawal, ewes received 480 IU of eCG. Cervical AI [18] was performed 54 ± 1 h after sponge withdrawal (14:00 p.m.), using an ovine AI gun (IMV, Instruments de Medicine Veterinaire, L’Aigle, France) and 0.25 mL French mini-straws. All of the inseminated ewes were *FecXR* allele non-carriers.

Births from AI were recorded on the farms throughout the year of the study. Fertility rate was the proportion of ewes lambing after AI, prolificacy was the number of lambs born per lambing, and fecundity rate was the number of lambs born per inseminated ewe.

### 2.5. Statistical Analyses

Semen quality, testicular dimensions, and reproductive performance after AI were evaluated statistically based on a multifactorial model that included the presence/absence of the *FecXR* allele (FecXR or ++ wild rams) and season as fixed effects in the Least-Squares Method of the GLM procedure in SPSS v.26 (IBM Corp., Released 2019). The seasons were defined based on the Northern Hemisphere Meteorological Season Division [19]. An ANOVA identified significant differences between genotypes and between seasons. The general representation of the model is as follows: y = xb + e, where y is N × 1 vector of records, b denotes the fixed effect in the model within the association matrix x, and e is the vector of residual effects. To test for significant differences between effect combinations, a post-hoc Fisher’s Least Significant Difference (LSD) test was used.

## 3. Results

### 3.1. Semen Quality

**Figure 1:**
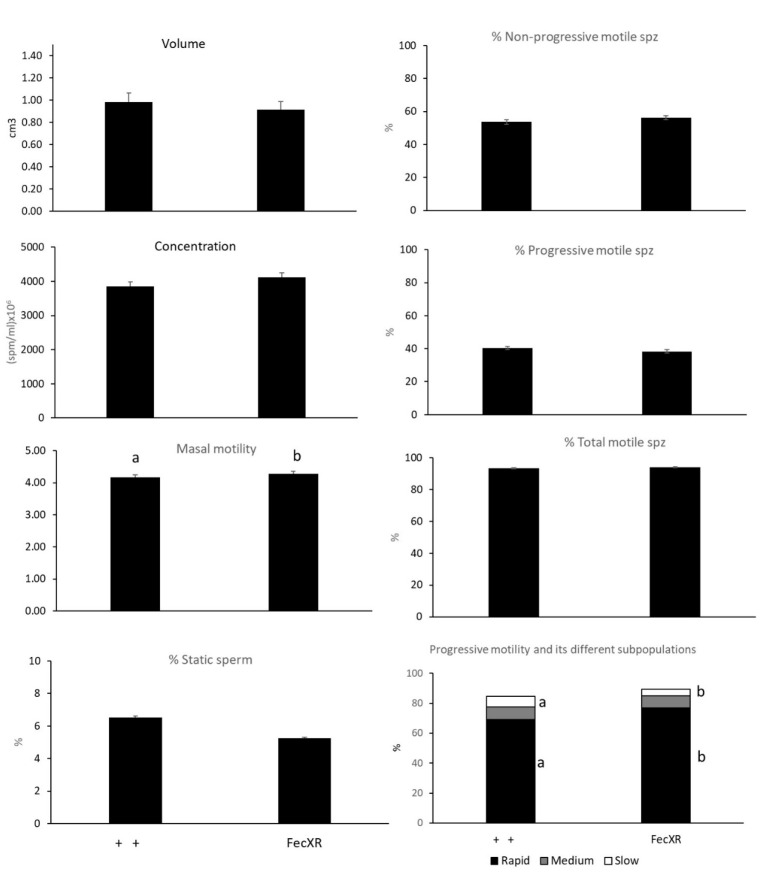
Seminal traits (mean ± S.E.M.) of Rasa Aragonesa rams of the wild genotype (+ +; *n* = 5) or those carrying the *FecXR* allele of the BMP15 gene (*n* = 10) (a,b indicate *p* < 0.05) (spz: spermatozoon). Values calculated from semen samples collected twice a week for one year.

Mean (±S.E.M.) sperm count (3762 ± 1060), ejaculate volume (0.93 ± 0.04 cm^3^) and semen concentration (4055 ± 100 × 10^6^) did not differ between the two genotypes, but concentration was significantly (*p* < 0.05) higher in summer than it was in autumn and winter (*p* < 0.05). Mass motility (4.26 ± 0.19) was significantly (*p* < 0.05) affected by the presence of the allele and season, with a significant (*p* = 0.01) interaction between effects. FecXR rams exhibited a higher mass motility (*p* < 0.05), a higher proportion of rapid sperm (*p* < 0.05), and a lower proportion of slow sperm (*p* < 0.0001) than did ++ rams (Figure 1). Mean proportion of NPM, PM, TM, and medium-speed sperm did not differ significantly between genotypes or among seasons.

The proportion of slow sperm was significantly (*p* < 0.05) lower in summer (Figure S1). In winter, FecXR rams tended to present a higher mass motility and a lower proportion of static sperm than did ++ rams (*p* < 0.10) (Table 1). Furthermore, FecXR rams had higher proportions of rapid sperm in spring (*p* < 0.10) and winter (*p* < 0.001), and lower proportions of slow sperm in spring (*p* < 0.05), summer (*p* < 0.05), and winter (*p* < 0.01) than did ++ rams (Table 1).

### 3.2. Testicular Measurements

SC (FecXR: 32.9 ± 0.6; ++: 31.8 ± 1.7 cm), TD (FecXR: 6.5 ± 0.2; ++: 6.2 ± 0.5 cm), and TL (FecXR: 9.3 ± 0.2; ++: 8.6 ± 0.7 cm) did not differ significantly between carriers and non-carriers of the *FecXR* allele; however, SC was highest in summer and winter (*p* < 0.05), TD was highest in summer and autumn (*p* < 0.01), TL was lowest in spring and winter (*p* < 0.05) (Table S1).

### 3.3. Reproductive Parameters

FecXR rams impregnated a significantly higher proportion (*p* < 0.05) of ewes (62.5 ± 2.5%) than did ++ rams (56.7 ± 2.9%), and fertility rates were lowest in spring and winter inseminations (Table S1). FecXR rams had significantly (*p* < 0.05) higher fertility rates than did ++ rams in winter inseminations, only (0.66 ± 0.08 vs. 0.40 ± 0.06%).

Prolificacy (FecXR: 1.73 ± 0.06; ++: 1.71 ± 0.06 lambs/lambing) and fecundity (FecXR: 1.10 ± 0.06; ++: 0.98 ± 0.06 lambs/ewe) did not differ significantly between FecXR and ++ rams, but differed significantly (*p* < 0.001) among seasons (Table S1).

## 4. Discussion

To our knowledge, this is the first study of the semen quality of rams carrying the *FecXR* allele. Ejaculate volume and sperm concentration did not differ significantly between the FecXR and wild rams in any season, which suggests that the production of seminal plasma or spermatogenesis are not affected by the *BMP15* gene. These results are similar to the observations of Kumar et al. [20] in Garole x Malpura rams carrying the *FecB* allele, and parallel the absence of differences in testicular size. Mass motility and the proportion of rapid sperm were significantly higher, and the proportion of slow sperm lower in the FecXR than they were in the wild-type rams.

Furthermore, ewes that had been inseminated with semen collected from FecXR rams had the highest mean annual fertility rate. Sperm motility and velocity are two of the most important aspects of semen quality because they are correlated with fertility [21]. In a study of Rasa Aragonesa breed at the same latitude as in our study, it has been reported that high-fertility rams produced a higher proportion of fast and linear spermatozoa than did low-fertility rams [22]. In Iberian deer, mean and maximum straight-line velocity of sperm and fertility are significantly correlated, and it appears that sperm swimming velocity is a main determinant of fertility in mammals [23]. Thus, it is likely that high mass motility and high proportion of rapid sperm contributed to the high fertility rates in FecXR rams. On the other hand, Lahoz et al. [24] did not detect significant differences between genotypes in a program that involved cervical insemination. Given the number of external factors that can affect the proportion of ewes that become pregnant after AI (year, farm, technician) [25] including weather [26] and climate [27], differences in the conditions at the time of experiments involving AI might have contributed to the presence or absence of differences between genotypes. Further study is needed to determine how external factors might influence the effect of *FecX* on reproductive parameters.

The finding that the highest fertility rate occurred in summer is similar to previous observations [28] in the same breed and at the same latitude as in our study, where the lowest AI fertility was between March and June, and the highest was in the first months of increasing daylength (July and August). Rasa Aragonesa is a reduced-seasonal anestrous breed [29], in which females exhibit an onset of the breeding season in July and a peak in ovulation rate in late August. Thus, our study confirms that summer is the peak breeding season for rams and ewes of this breed.

Differences in the pregnancy rates related to polymorphisms of the *BMP15* gene have been reported by Sun et al. [30], who found that Chinese Holstein bulls of the CT genotype had a significantly lower sperm motility than did bulls of the CC or TT genotypes. In sheep, Chen et al. [13] reported the expression of *BMP15* in the epididymis of rams, which was significantly higher in a less-fecund breed (Sunite) than it was in a high-fecundity breed (Small Tail Han). Possibly, the expression level of *BMP15* and fecundity in rams are negatively correlated. Garole × Malpura rams that carry the *FecB* genotype had a significantly higher proportion of rapid motile sperm and with higher linearity, and a higher FSH concentration than did the wild type [15].

In our study, testicular dimensions did not differ significantly between rams that carried the *FecXR* allele and those that did not. Rasa Aragonesa light lambs that did or did not carry the *FecXR* allele did not exhibit significant differences in birth weight, growth rate, or carcass quality [31]; moreover, it appears that *FecXR* allele may not influence testicular morphology or fleece weight at 13 months of age in carrier Romney rams [32]. The absence of differences in testicular measurements between genotypes parallels the lack of differences in sperm volume and concentration, which are highly correlated to testicle size [33].

Season had a significant effect on testicular measurements, which was similar to the effects reported by Avdi et al. [34] in Chios and Serres rams. Similarly, Chios and Friesian rams had semen characteristics that were generally better in summer and peaked in quality in autumn [35]. Although seasonal variations in reproductive traits in sheep are less marked in rams than they are in ewes, the consequences of the non-reproductive season are smaller testicular volume and diameter, lower semen quality, and hormone profiles that differ from those in the breeding season [36]. Photoperiod is the key environmental signal that dictates the timing of the reproductive cycle of the ram [37], which is synchronized through changes in daily melatonin secretion [38]. Rams exhibit a seasonal decrease in sexual behavior and spermatogenesis at about the time that ewes are in sexual rest, but with a 1- to 2-month advance in phase [39].

## 5. Conclusions

In conclusion, this study demonstrated that carriers of the *FecXR* allele produce good-quality semen throughout the year, and corresponded with an improvement in some sperm characteristics—particularly mass motility and the proportion of rapid sperm—along with an interaction effect with season. In addition, the ability to pass the allele to their female offspring, through the insemination of wild type, non-carriers ewes, makes these males especially valuable for AI to produce prolific ewes.

## Figures and Tables

**Table 1:** Seminal traits (mean ± S.E.M.) of Rasa Aragonesa rams of the wild genotype (++; *n* = 5) or carrying the *FecXR* allele of the *BMP15* gene (*n* = 10) (* indicate differences *p* < 0.10 within season) (** indicate significant differences at *p* < 0.05 within season). Values calculated from semen samples collected twice a week for one year. NPM: non-progressive motile sperm; PM: progressive motile sperm; TM: total motile sperm.

	Spring		Summer		Autumn		Winter	
	++	FecXR	++	FecXR	++	FecXR	++	FecXR
Sperm count (×10^6^)	4020 ± 311	3665 ± 533	4381 ± 91	4456 ± 364	3465 ± 553	3297 ± 375	3352 ± 338	3525 ± 458
Volume (cm^3^)	0.91 ± 0.04	0.86 ± 0.10	1.10 ± 0.02	0.99 ± 0.10	0.92 ± 0.09	0.84 ± 0.09	0.97 ± 0.03	0.97 ± 0.11
Concentration (×10^6^)	4420 ± 140	4163 ± 256	3987 ± 146	4572 ± 209	3713 ± 252	4002 ± 242	3452 ± 241	3609 ± 143
Mass motility (0–5)	4.30 ± 0.00	4.28 ± 0.01	4.27 ± 0.03	4.29 ± 0.01	4.29 ± 0.01	4.28 ± 0.02	3.83 ± 0.42 *	4.30 ± 0.00
% Static spz	3.43 ± 0.88	5.65 ± 2.24	8.33 ± 3.34	4.93 ± 0.90	5.89 ± 0.95	6.79 ± 1.80	7.44 ± 0.29	5.48 ± 0.61
% NPM spz	57.93 ± 0.88	54.99 ± 1.84	52.78 ± 3.58	53.93 ± 2.32	54.78 ± 3.16	58.55 ± 2.45	50.67 ± 4.10	57.57 ± 1.19
% PM spz	41.66 ± 3.01	40.71 ± 2.76	38.89 ± 4.08	41.13 ± 2.09	39.33 ± 2.22	34.67 ± 1.72	41.89 ± 4.37	36.95 ± 1.41
% TM spz	96.41 ± 1.04	93.87 ± 2.21	91.67 ± 3.34	95.07 ± 0.90	94.11 ± 0.95	93.21 ± 1.80	92.56 ± 0.29	94.38 ± 0.65
% Rapid spz	69.97 ± 5.27	75.50 ± 3.83	69.78 ± 8.42 *	80.23 ± 2.21	76.33 ± 2.96	75.09 ± 3.68	60.89 ± 2.63 **	76.52 ± 2.69
% Medium spz	6.31 ± 0.94	8.43 ± 1.16	8.33 ± 1.20	7.17 ± 1.17	8.11 ± 1.46	8.33 ± 1.14	10.33 ± 2.19	8.52 ± 0.89
% Slow spz	18.69 ± 5.26 **	8.95 ± 1.79	11.78 ± 4.11 **	6.23 ± 0.54	9.00 ± 3.02	8.94 ± 1.83	20.67 ± 1.86 **	8.48 ± 1.58

## Supplementary Materials

The following are available online at https://www.mdpi.com/2076-2615/10/9/1628/s1, Figure S1: Annual seminal traits (mean ± S.E.M.) of Rasa Aragonesa rams (a,b indicate *p* < 0.05) (spz: spermatozoon). Values calculated from semen samples collected twice a week for one year, Table S1: Mean (± S.E.M.) testicular measurements and reproductive traits of Rasa Aragonesa rams (*n* = 15) (a,b,c indicate significant differences *p* < 0.05). Values calculated from semen samples collected twice a week for one year.

## References

[1] Vadhana E., Santhosh A., Pooja G.S., Vani A., Kumar S. (2019). FecB: A major gene governing fecundity in sheep. J. Entomol. Zool. Stud..

[2] Galloway S.M., McNatty K.P., Cambridge L.M., Laitinen M.P.E., Juengel J.L., Jokiranta T.S., McLaren R.J., Luiro K., Dodds K.G., Montgomery G.W. (2000). Mutations in an oocyte-derived growth factor gene (BMP15) cause increased ovulation rate and infertility in a dosage-sensitive manner. Nat. Genet..

[3] Moore R.K., Shimasaki S. (2005). Molecular biology and physiological role of the oocyte factor, BMP-15. Mol. Cell. Endocrinol..

[4] Montgomery G.W., Galloway S.M., Davis G.H., McNatty K.P. (2001). Genes controlling ovulation rate in sheep. Reproduction.

[5] Hanrahan J.P., Gregan S.M., Mulsant P., Mullen M., Davis G.H., Powell R., Galloway S.M. (2004). Mutations in the genes for oocyte-derived growth factors GDF9 and BMP15 are associated with both increased ovulation rate and sterility in Cambridge and Belclare sheep (Ovis aries). Biol. Reprod..

[6] Davis G.H. (2005). Major genes affecting ovulation rate in sheep. Genet. Sel. Evol..

[7] Bodin L., Di Pasquale E., Fabre S., Bontoux M., Monget P., Persani L., Mulsant P.A. (2007). A novel mutation in the bone morphogenetic protein 15 gene causing defective protein secretion is associated with both increased ovulation rate and sterility in Lacaune sheep. Endocrinology.

[8] Monteagudo L.V., Ponz R., Tejedor M.T., Laviña A., Sierra I. (2009). A 17 bp deletion in the Bone Morphogenetic Protein 15 (BMP15) gene is associated to increased prolificacy in the Rasa Aragonesa sheep breed. Anim. Reprod. Sci..

[9] Demars J., Fabre S., Sarry J., Rossetti R., Gilbert H., Persani L., Tosser-Klopp G., Mulsant P., Nowak Z., Drobik W. (2013). Genome-wide association studies identify two novel BMP15 mutations responsible for an atypical hyperprolificacy phenotype in sheep. PLoS Genet..

[10] Otsuka F., McTavish K.J., Shimasaki S. (2011). Integral role of GDF-9 and BMP-15 in ovarian function. Mol. Reprod. Dev..

[11] ANGRA. https://www.rasaaragonesa.com.

[12] Sierra I. (1992). La raza ovina Rasa Aragonesa: Caracteres morfológicos y productivos. Anim. Genet. Resour. Inf..

[13] Chen W., Tian Z., Ma L., Gan S., Sun W., Chu M. (2020). Expression analysis of BMPR1B, BMP15, GDF9, Smad1, Smad5, and Smad9 in rams with different fecundity. Pak. J. Zool..

[14] Lahoz B., Blasco M.E., Sevilla E., Folch J., Roche A., Quintin F.J., Martínez-Royo A., Galeote A.I., Calvo J.H., Fantova E. Fertility of Rasa Aragonesa rams carrying or not the FecXR allele of BMP15 gene when used in artificial insemination. Proceedings of the European Association for Animal Production (EAAP).

[15] Kumar D., Joshi A., Naqvi S.M.K., Kumar S., Mishrac A.K., Maurya V.P., Arora A.L., Mittal J.P., Singh V.K. (2007). Sperm motion characteristics of Garole × Malpura sheep evolved in a semi-arid tropical environment through introgression of FecB gene. Anim. Reprod. Sci..

[16] Evans G., Maxwell W.M.C., Salamon S. (1987). Handling and examination semen. Salamon’S Artificial Insemination of Sheep and Goats.

[17] Palacín I., Vicente-Fiel S., Santolaria P., Yániz J.L. (2013). Standardization of CASA sperm motility assessment in the ram. Small Rumin. Res..

[18] Macías A., Ferrer L.M., Ramos J.J., Lidón I., Rebollar R., Lacasta D., Tejedor M.J. (2017). Technical Note: A new device for cervical insemination of sheep—Design and field test. J. Anim. Sci..

[19] Trenberth K.E. (1983). What are the Seasons?. Bull. Am. Meteorol. Soc..

[20] Kumar D., Naqvi S.M.K., Kumar S. (2012). Sperm motion characteristics of FecBBB and FecBB + Garole × Malpura rams during the non-breeding season under hot semi-arid environment. Livest. Sci..

[21] Aitkin R.J., Gagnon C. (1990). Motility parameters and fertility. Control of Sperm Motility: Biological and Clinical Aspects.

[22] Yániz J.L., Palacín I., Vicente-Fiel S., Sánchez-Nadal J.A., Santolaria P. (2015). Sperm population structure in high and low field fertility rams. Anim. Reprod. Sci..

[23] Gomendio M., Roldán E.R.S. (2008). Implications of diversity in sperm size and function for sperm competition and fertility. Int. J. Dev. Biol..

[24] Lahoz B., Alabart J.L., Jurado J.J., Calvo J.H., Martínez-Royo A., Fantova E., Folch J. (2011). Effect of the FecXR polymorphism in the bone morphogenetic protein 15 gene on natural or equine chorionic gonadotropin-induced ovulation rate and litter size in Rasa Aragonesa ewes and implications for on-farm application. J. Anim. Sci..

[25] Anel L., Kaabi M., Abroug B., Alvarez M., Anel E., Boixo J.C., de la Fuente L.F., Paz P. (2005). Factors influencing the success of vaginal and laparoscopic artificial insemination in Churra ewes: A field assay. Theriogenology.

[26] Palacios C., Abecia J.A. (2015). Meteorological variables affect fertility rate afterintrauterine artificial insemination in sheep in a seasonal-dependent manner: A 7-year study. Int. J. Biometeorol..

[27] Abecia J.A., Máñez J., Macias A., Laviña A., Palacios C. (2017). Climate zone influences the effect of temperature on the day of artificial insemination on fertility in two Iberian sheep breeds. J. Anim. Behav. Biometeorol..

[28] Palacín I., Yániz J.L., Fantova E., Blasco M.E., Quintín-Casorrán F.J., Sevilla-Mur E., Santolaria P. (2012). Factors affecting fertility after cervical insemination with cooled semen in meat sheep. Anim. Reprod. Sci..

[29] Forcada F., Abecia J.A., Sierra I. (1992). Seasonal changes in oestrous activity and ovulation rate in Rasa Aragonesa ewes maintained at two different body condition levels. Small Rumin. Res..

[30] Sun L.P., Song Y.P., Du Q.Z., Song L.W., Tian Y.Z., Zhang S.L., Hua G.H., Yang L.G. (2014). Polymorphisms in the bone morphogenetic protein 15 gene and their effect on sperm quality traits in Chinese Holstein bulls. Genet. Mol. Res..

[31] Roche A., Ripoll G., Joy M., Folch J., Panea B., Calvo J.H., Alabart J.L. (2012). Effects of the FecX R allele of BMP15 gene on the birth weight, growth rate and carcass quality of Rasa Aragonesa light lambs. Small Rumin. Res..

[32] Davis G.H., Dodds K.G., McEwan J.C., Fennessy P.F. (1993). Liveweight, fleece weight and prolificacy of Romney ewes carrying the Inverdale prolificacy gene (FecXI) located on the X-chromosome. Livest. Prod. Sci..

[33] Kheradmand A., Babaei H., Abshenas J. (2006). Comparative evaluation of the effect of antioxidants on the chilled-stored ram semen. Iran. J. Vet. Res..

[34] Avdi M., Banos G., Stefos K., Chemineau P. (2004). Seasonal variation in testicular volume and sexual behavior of Chios and Serres rams. Theriogenology.

[35] Karagiannidis A., Varsakeli S., Alexopoulos C., Amarantidis I. (2000). Seasonal variation in semen characteristics of Chios and Friesian rams in Greece. Small Rumin. Res..

[36] Casao A., Vega S., Palacín I., Pérez-Pe R., Laviña A., Quintín F.J., Sevilla E., Abecia J.A., Cebrián-Pérez J.A., Forcada F. (2010). Effects of Melatonin Implants During Non-Breeding Season on Sperm Motility and Reproductive Parameters in Rasa Aragonesa Rams. Reprod. Dom. Anim..

[37] Lincoln G.A., Short V. (1980). Seasonal Breeding: Nature’s Contraceptive. Recent Prog. Horm. Res..

[38] Malpaux B., Viguié C., Skinner D.C., Thiéry J.C., Pelletier J., Chemineau P. (1996). Seasonal breeding in sheep: Mechanism of action of melatonin. Anim. Reprod. Sci..

[39] Lincoln G.A., Serio M. (1989). Significance of seasonal cycles in prolactin secretion in male mammals. Perspectives in Andrology.

