# Supplementary material for: Semen Quality of Rasa Aragonesa Rams Carrying the FecXR Allele of the BMP15 Gene
Source: Animals (Basel). 2020 Sep 11;10(9):1628. doi: 10.3390/ani10091628 (PMC7552265; doi:10.3390/ani10091628)
Supplement: Supplementary file 1 [file animals-10-01628-s001.pdf]

animals

Supplementary Materials: Semen Quality of Rasa Aragonesa Rams Carrying the *FecXR* Allele of the *BMP15* Gene

José Alfonso Abecia [1,*], Ángel Macías [2], Adriana Casao [1], Clara Burillo [2], Elena Martín [2], Rosaura Pérez-Pé [1] and Adolfo Laviña [2]

[1] University Institute of Research in Environmental Sciences of Aragon (IUCA), University of Zaragoza, Miguel Servet, 177, 50013 Zaragoza, Spain; adriana@unizar.es (A.C.); rosperez@unizar.es (R.P.-P.)
[2] National Association of Rasa Aragonesa Breeders (ANGRA), Cabañera Real, s/n, 50800 Zuera, Spain; angel@rasaaragonesa.com (A.M.); aabecia0@gmail.com (C.B.); jabecia1@alumno.uned.es (E.M.); adolfo@rasaaragonesa.com (A.L.)
* Correspondence: alf@unizar.es

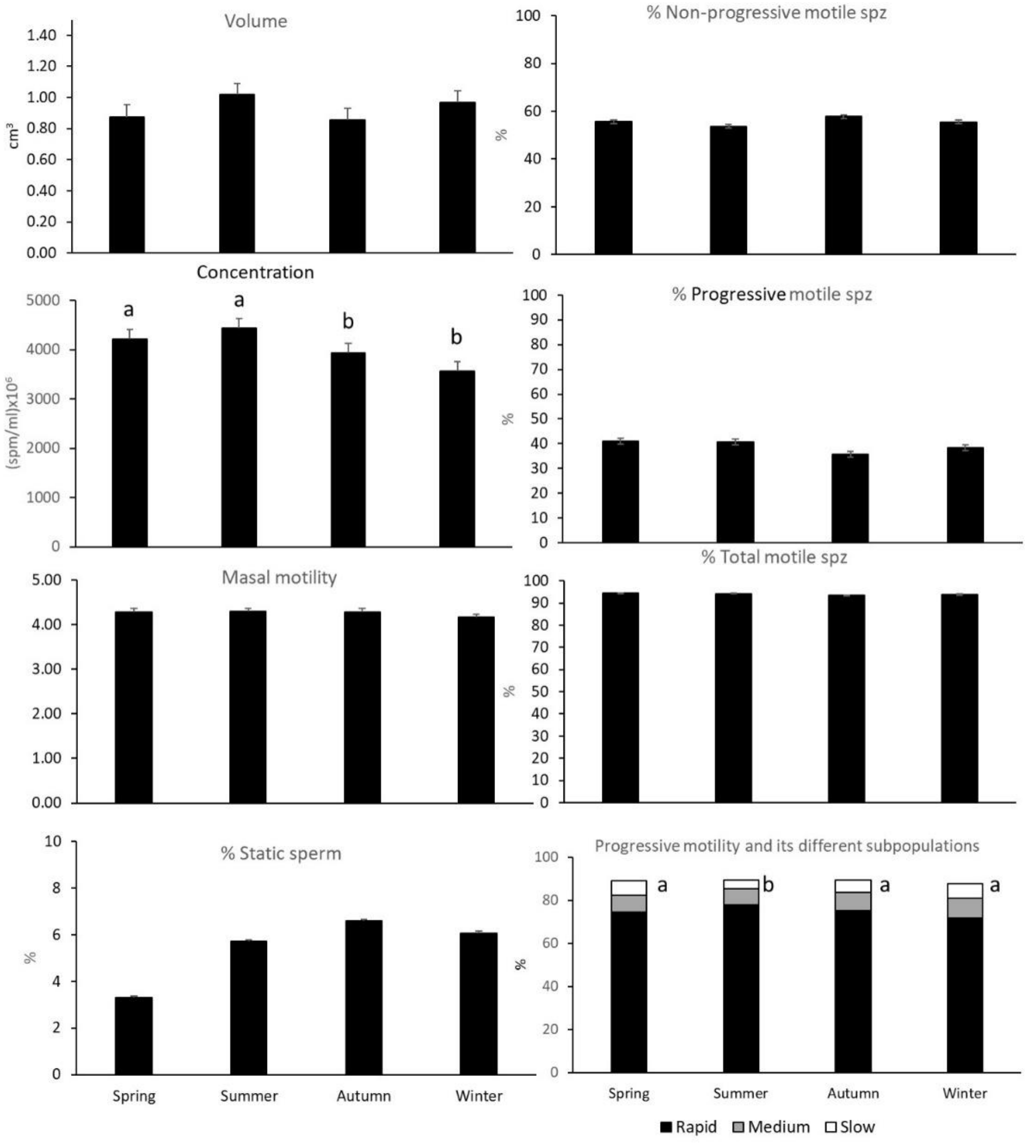

Figure S1. Annual seminal traits (mean ± S.E.M.) of Rasa Aragonesa rams (a,b indicate $p < 0.05$) (spz: spermatozoon). Values calculated from semen samples collected twice a week for one year.

Table 1. Mean (± S.E.M.) testicular measurements and reproductive traits of Rasa Aragonesa rams (n = 15) (a,b,c indicate significant differences $p < 0.05$). Values calculated from semen samples collected twice a week for one year.

	**Spring**	**Summer**	**Autumn**	**Winter**
Scrotal circumference (cm)	33.1 ± 0.7 [a]	34.3 ± 0.4 [b]	33.8 ± 0.4 [a]	34.8 ± 0.6 [b]
Testicular diameter (cm)	6.8 ± 0.2 [a]	7.0 ± 0.1 [b]	6.7 ± 0.1 [a]	6.7 ± 0.9 [a]
Testicular length (cm)	9.0 ± 0.8 [a]	10.3 ± 0.2 [b]	9.7 ± 0.5 [b]	9.2 ± 0.2 [a]
Fertility (%)	52.9 ± 0.05 [a]	65.9 ± 0.02 [b]	59.2 ± 0.04 [a]	50.6 ± 0.06 [c]
Prolificacy (lambs/lambing)	1.63 ± 0.09 [a]	1.88 ± 0.04 [b]	1.65 ± 0.07 [a]	1.30 ± 0.18 [c]
Fecundity (lambs/ewe)	0.8 6± 0.09 [a]	1.25 ± 0.06 [b]	0.97 ± 0.07 [a]	0.59 ± 0.09 [c]
